# *N*′-(2-Methyl-3-phenyl­allyl­idene)nicotinohydrazide monohydrate

**DOI:** 10.1107/S1600536809023368

**Published:** 2009-06-20

**Authors:** R. Archana, A. Manimekalai, N. Saradhadevi, A. Thiruvalluvar, R. J. Butcher

**Affiliations:** a PG Research Department of Physics, Rajah Serfoji Government College (Autonomous), Thanjavur 613 005, Tamil Nadu, India; b Department of Chemistry, Annamalai University, Annamalai Nagar 608 002, Tamilnadu, India; c Department of Chemistry, Howard University, 525 College Street NW, Washington, DC 20059, USA

## Abstract

The asymmetric unit of the title compound, C_16_H_15_N_3_O·H_2_O, contains an *N*′-(2-methyl-3-phenyl­allyl­idene)nicotino­hydra­zide mol­ecule and a water solvent mol­ecule. The dihedral angle between the pyridine ring and the phenyl ring is 47.26 (5)°. Inter­molecular O—H⋯N, O—H⋯O, N—H⋯O and C—H⋯O hydrogen bonds are found in the crystal structure. Furthermore, C—H⋯π inter­actions involving the pyridine and phenyl rings are also found.

## Related literature

For a related crystal structure, see: Bao (2008 ▶). For chemical and biological applications of related compounds, see: Moraweck *et al.* (1997 ▶); Kwon *et al.* (1996 ▶); Lee *et al.* (1999 ▶).
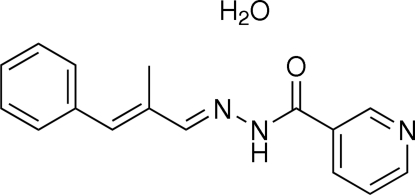


## Experimental

### 

#### Crystal data

C_16_H_15_N_3_O·H_2_O
*M* *_r_* = 283.33
Monoclinic, 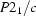

*a* = 9.6821 (4) Å
*b* = 9.4178 (4) Å
*c* = 16.0958 (6) Å
β = 98.250 (4)°
*V* = 1452.49 (10) Å^3^
*Z* = 4
Mo *K*α radiation
μ = 0.09 mm^−1^
*T* = 110 K
0.51 × 0.42 × 0.36 mm

#### Data collection

Oxford Diffraction Gemini R diffractometer
Absorption correction: multi-scan (*CrysAlis RED*; Oxford Diffraction, 2008 ▶) *T*_min_ = 0.938, *T*_max_ = 1.000 (expected range = 0.909–0.969)
10476 measured reflections
4824 independent reflections
3467 reflections with *I* > 2σ(*I*)
*R*_int_ = 0.021

#### Refinement

*R*[*F* ^2^ > 2σ(*F* ^2^)] = 0.042
*wR*(*F* ^2^) = 0.119
*S* = 1.02
4824 reflections
203 parameters
H atoms treated by a mixture of independent and constrained refinement
Δρ_max_ = 0.40 e Å^−3^
Δρ_min_ = −0.22 e Å^−3^

### 

Data collection: *CrysAlis CCD* (Oxford Diffraction, 2008 ▶); cell refinement: *CrysAlis RED* (Oxford Diffraction, 2008 ▶); data reduction: *CrysAlis RED*; program(s) used to solve structure: *SHELXS97* (Sheldrick, 2008 ▶); program(s) used to refine structure: *SHELXL97* (Sheldrick, 2008 ▶); molecular graphics: *ORTEP-3* (Farrugia, 1997 ▶); software used to prepare material for publication: *PLATON* (Spek, 2009 ▶).

## Supplementary Material

Crystal structure: contains datablocks global, I. DOI: 10.1107/S1600536809023368/wn2333sup1.cif

Structure factors: contains datablocks I. DOI: 10.1107/S1600536809023368/wn2333Isup2.hkl

Additional supplementary materials:  crystallographic information; 3D view; checkCIF report

## Figures and Tables

**Table 1:** Hydrogen-bond geometry (Å, °)

*D*—H⋯*A*	*D*—H	H⋯*A*	*D*⋯*A*	*D*—H⋯*A*
O1*W*—H1*W*⋯N1^i^	0.917 (17)	1.987 (17)	2.9033 (11)	177.3 (16)
O1*W*—H2*W*⋯O7	0.851 (17)	2.161 (17)	2.9089 (10)	146.5 (15)
O1*W*—H2*W*⋯N9	0.851 (17)	2.507 (17)	3.2233 (11)	142.5 (14)
N8—H8⋯O1*W*^ii^	0.882 (14)	2.029 (14)	2.8925 (12)	166.0 (13)
C2—H2⋯O7^i^	0.95	2.54	3.4021 (12)	151
C10—H10⋯O1*W*^ii^	0.95	2.59	3.3781 (13)	140
C13—H13*B*⋯O1*W*	0.98	2.55	3.3815 (14)	143
C26—H26⋯O7^iii^	0.95	2.54	3.4771 (13)	170
C13—H13*C*⋯*Cg*1^iv^	0.98	2.72	3.5630 (13)	144
C5—H5⋯*Cg*2^v^	0.95	2.57	3.4378 (11)	152

Symmetry codes: (i) 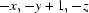; (ii) 
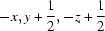
; (iii) 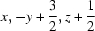; (iv) 
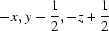
; (v) 
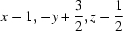
. *Cg*1 and *Cg*2 are the centroids of the pyridine and benzene rings, respectively.

**Fig. 1.**
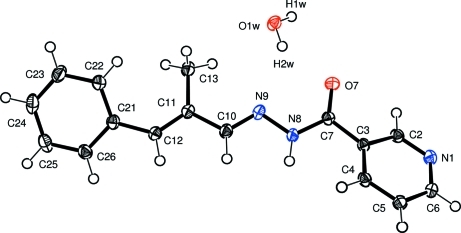
The molecular structure of the asymmetric unit, showing the atom-numbering scheme and displacement ellipsoids drawn at the 50% probability level. H atoms are shown as small spheres of arbitrary radius.

**Fig. 2.**
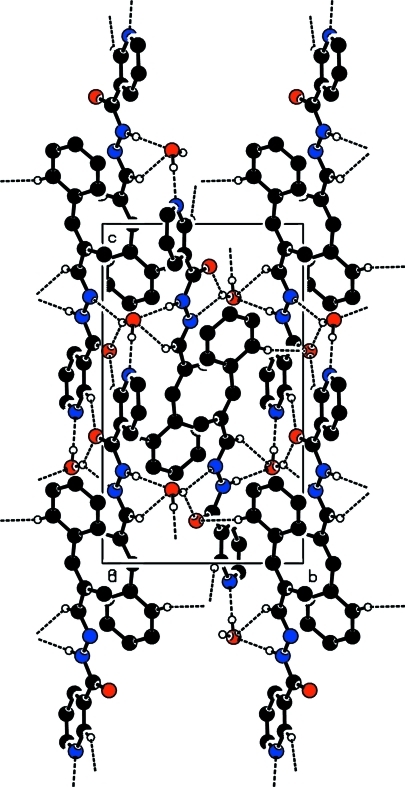
The packing of the title compound, viewed down the a axis. Dashed lines indicate hydrogen bonds. H atoms not involved in hydrogen bonding have been omitted.

## supplementary crystallographic information

### Comment

Cinnamaldehyde is of importance in the manufacture of fine chemicals, particularly with regard to fragrances and flavorings (Moraweck *et al.*, 1997). 2'-Hydroxycinnamaldehyde was isolated from the stem bark of Cinnamomum cassia and reported to have an inhibitory effect on farnesyl protein transferase activity; it also inhibited the proliferation of several human cancer cell lines including breast, leukemia, ovarian, lung, and colon tumor cells. Nicotinic hydrazide is used as a medicine for key diseases such as leprosy (Hansen's disease), typhoid and tuberculosis (Kwon *et al.*, 1996; Lee *et al.*, 1999). As part of our research, we have synthesized the title compound and report its crystal structure here. Bao (2008) has reported a related crystal structure, viz. *N*'-(3-phenylallylidene)isonicotinohydrazide.

The molecular structure of the asymmetric unit is shown in Fig. 1. The dihedral angle between the pyridine ring and the phenyl ring is 47.26 (5)°. Intermolecular O—H···N, O—H···O, N—H···O and C—H···O hydrogen bonds are found in the crystal structure. Furthermore, a C13—H13C···π interaction involving the pyridine (N1—C6) ring and a C5—H5···π interaction involving the phenyl (C21—C26) ring are also found.

### Experimental

Sodium hydroxide (0.4 g, 0.01 mol) in a stoppered conical flask was kept in an ice-cold environment. Ethanol (40 ml) was added to dissolve it and the mixture was stirred continuously using a magnetic stirrer. An equimolar quantity of nicotinic hydrazide (1.371 g, 0.01 mol) and α-methyl-*trans*-cinnamaldehyde (1.461 g, 0.01 mol) was added to this mixture. The stirring was continued for 5 h in ice-cold conditions. The mixture was kept overnight in a refrigerator. The mixture was then allowed to stand for four days under normal conditions. A yellow solid was obtained. This was filtered, washed and recrystallized from ethanol. Yield 2.2 g, 48.47%.

### Refinement

H8 attached to N8, and H1W and H2W attached to O1W were located in a difference Fourier map and refined isotropically. The remaining H atoms were positioned geometrically and allowed to ride on their parent atoms, with C—H = 0.95 and 0.98 Å for C*sp*^2^ and methyl H atoms, respectively. *U*_iso_(H) = *xU*_eq_(C), where *x* = 1.5 for methyl H atoms and 1.2 for other C-bound H atoms.

### Crystal data

**Table:**

C_16_H_15_N_3_O·H_2_O	*F*(000) = 600
*M**_r_* = 283.33	*D*_x_ = 1.296 Mg m^−^^3^
Monoclinic, *P*2_1_/*c*	Melting point: 400(2) K
Hall symbol: -P 2ybc	Mo *K*α radiation, λ = 0.71073 Å
*a* = 9.6821 (4) Å	Cell parameters from 4977 reflections
*b* = 9.4178 (4) Å	θ = 4.6–32.6°
*c* = 16.0958 (6) Å	µ = 0.09 mm^−^^1^
β = 98.250 (4)°	*T* = 110 K
*V* = 1452.49 (10) Å^3^	Block, colourless
*Z* = 4	0.51 × 0.42 × 0.36 mm

### Data collection

**Table:**

Oxford Diffraction Gemini R diffractometer	4824 independent reflections
Radiation source: fine-focus sealed tube	3467 reflections with *I* > 2σ(*I*)
graphite	*R*_int_ = 0.021
Detector resolution: 10.5081 pixels mm^-1^	θ_max_ = 32.8°, θ_min_ = 4.7°
φ and ω scans	*h* = −14→13
Absorption correction: multi-scan (*CrysAlis RED*; Oxford Diffraction, 2008)	*k* = −12→14
*T*_min_ = 0.938, *T*_max_ = 1.000	*l* = −17→24
10476 measured reflections	

### Refinement

**Table:**

Refinement on *F*^2^	Primary atom site location: structure-invariant direct methods
Least-squares matrix: full	Secondary atom site location: difference Fourier map
*R*[*F*^2^ > 2σ(*F*^2^)] = 0.042	Hydrogen site location: inferred from neighbouring sites
*wR*(*F*^2^) = 0.119	H atoms treated by a mixture of independent and constrained refinement
*S* = 1.02	*w* = 1/[σ^2^(*F*_o_^2^) + (0.0703*P*)^2^] where *P* = (*F*_o_^2^ + 2*F*_c_^2^)/3
4824 reflections	(Δ/σ)_max_ = 0.001
203 parameters	Δρ_max_ = 0.40 e Å^−^^3^
0 restraints	Δρ_min_ = −0.22 e Å^−^^3^

### Special details

**Table:**

Geometry. Bond distances, angles *etc*. have been calculated using the rounded fractional coordinates. All su's are estimated from the variances of the (full) variance-covariance matrix. The cell e.s.d.'s are taken into account in the estimation of distances, angles and torsion angles
Refinement. Refinement of *F*^2^ against ALL reflections. The weighted *R*-factor *wR* and goodness of fit *S* are based on *F*^2^, conventional *R*-factors *R* are based on *F*, with *F* set to zero for negative *F*^2^. The threshold expression of *F*^2^ > 2σ(*F*^2^) is used only for calculating *R*-factors(gt) *etc*. and is not relevant to the choice of reflections for refinement. *R*-factors based on *F*^2^ are statistically about twice as large as those based on *F*, and *R*- factors based on ALL data will be even larger.

### Fractional atomic coordinates and isotropic or equivalent isotropic displacement parameters (Å^2^)

**Table:**

	*x*	*y*	*z*	*U*_iso_*/*U*_eq_	
O7	−0.01746 (7)	0.46732 (8)	0.12555 (4)	0.0228 (2)	
N1	−0.32793 (9)	0.63377 (9)	−0.05360 (5)	0.0216 (2)	
N8	−0.09368 (8)	0.60130 (10)	0.22771 (5)	0.0191 (2)	
N9	0.01977 (8)	0.56186 (9)	0.28577 (5)	0.0201 (2)	
C2	−0.22360 (10)	0.59151 (11)	0.00496 (6)	0.0189 (3)	
C3	−0.23120 (9)	0.59362 (10)	0.09091 (6)	0.0163 (2)	
C4	−0.35482 (10)	0.63912 (10)	0.11676 (6)	0.0186 (3)	
C5	−0.46492 (10)	0.68046 (11)	0.05655 (6)	0.0205 (3)	
C6	−0.44662 (10)	0.67645 (11)	−0.02681 (6)	0.0212 (3)	
C7	−0.10495 (10)	0.54718 (10)	0.14926 (6)	0.0173 (2)	
C10	0.02233 (10)	0.61992 (11)	0.35825 (6)	0.0198 (3)	
C11	0.13277 (10)	0.58878 (10)	0.42696 (6)	0.0186 (2)	
C12	0.12207 (10)	0.65038 (10)	0.50151 (6)	0.0201 (3)	
C13	0.24871 (12)	0.49192 (13)	0.41017 (7)	0.0289 (3)	
C21	0.21352 (10)	0.63767 (10)	0.58181 (6)	0.0189 (3)	
C22	0.30403 (11)	0.52316 (11)	0.60385 (6)	0.0246 (3)	
C23	0.39472 (12)	0.52335 (13)	0.67868 (7)	0.0295 (3)	
C24	0.39566 (11)	0.63518 (14)	0.73431 (6)	0.0301 (3)	
C25	0.30184 (11)	0.74592 (13)	0.71563 (7)	0.0290 (3)	
C26	0.21206 (10)	0.74683 (12)	0.64052 (6)	0.0225 (3)	
O1W	0.23612 (8)	0.34636 (8)	0.21718 (5)	0.0221 (2)	
H2	−0.14001	0.55834	−0.01294	0.0227*	
H4	−0.36389	0.64191	0.17472	0.0223*	
H5	−0.55094	0.71084	0.07247	0.0246*	
H6	−0.52209	0.70546	−0.06764	0.0254*	
H8	−0.1500 (15)	0.6670 (15)	0.2429 (8)	0.035 (4)*	
H10	−0.04946	0.68455	0.36710	0.0238*	
H12	0.04363	0.71085	0.50187	0.0241*	
H13A	0.33627	0.52239	0.44365	0.0434*	
H13B	0.25826	0.49565	0.35041	0.0434*	
H13C	0.22731	0.39448	0.42547	0.0434*	
H22	0.30311	0.44436	0.56693	0.0295*	
H23	0.45685	0.44589	0.69188	0.0354*	
H24	0.45988	0.63623	0.78482	0.0361*	
H25	0.29922	0.82139	0.75454	0.0348*	
H26	0.14816	0.82318	0.62863	0.0270*	
H1W	0.2677 (16)	0.3540 (16)	0.1663 (11)	0.054 (5)*	
H2W	0.1656 (18)	0.4012 (18)	0.2096 (10)	0.055 (5)*	

### Atomic displacement parameters (Å^2^)

**Table:**

	*U*^11^	*U*^22^	*U*^33^	*U*^12^	*U*^13^	*U*^23^
O7	0.0208 (3)	0.0255 (4)	0.0221 (4)	0.0058 (3)	0.0028 (3)	−0.0028 (3)
N1	0.0238 (4)	0.0249 (4)	0.0160 (4)	0.0008 (3)	0.0024 (3)	−0.0009 (3)
N8	0.0159 (4)	0.0244 (4)	0.0161 (4)	0.0031 (3)	−0.0010 (3)	−0.0029 (3)
N9	0.0176 (4)	0.0237 (4)	0.0179 (4)	0.0010 (3)	−0.0015 (3)	0.0020 (3)
C2	0.0186 (4)	0.0214 (5)	0.0174 (4)	−0.0001 (4)	0.0048 (3)	−0.0017 (4)
C3	0.0158 (4)	0.0167 (4)	0.0161 (4)	−0.0016 (3)	0.0018 (3)	−0.0012 (3)
C4	0.0184 (4)	0.0225 (5)	0.0154 (4)	−0.0014 (4)	0.0040 (3)	−0.0026 (3)
C5	0.0165 (4)	0.0239 (5)	0.0210 (5)	0.0011 (4)	0.0021 (3)	−0.0034 (4)
C6	0.0196 (5)	0.0239 (5)	0.0188 (5)	0.0014 (4)	−0.0017 (3)	−0.0014 (4)
C7	0.0162 (4)	0.0183 (4)	0.0173 (4)	−0.0015 (3)	0.0025 (3)	−0.0005 (3)
C10	0.0175 (4)	0.0213 (5)	0.0198 (4)	−0.0004 (4)	−0.0003 (3)	0.0000 (4)
C11	0.0178 (4)	0.0189 (4)	0.0186 (4)	−0.0009 (3)	0.0005 (3)	0.0017 (4)
C12	0.0181 (4)	0.0211 (5)	0.0200 (4)	0.0016 (4)	−0.0010 (3)	−0.0008 (4)
C13	0.0297 (6)	0.0374 (6)	0.0186 (5)	0.0116 (5)	0.0001 (4)	−0.0012 (4)
C21	0.0170 (4)	0.0216 (5)	0.0180 (4)	−0.0021 (4)	0.0018 (3)	0.0003 (4)
C22	0.0309 (5)	0.0241 (5)	0.0182 (5)	0.0046 (4)	0.0013 (4)	0.0002 (4)
C23	0.0291 (5)	0.0381 (6)	0.0206 (5)	0.0086 (5)	0.0012 (4)	0.0069 (4)
C24	0.0252 (5)	0.0476 (7)	0.0166 (5)	−0.0022 (5)	0.0003 (4)	0.0008 (4)
C25	0.0274 (5)	0.0393 (7)	0.0205 (5)	−0.0043 (5)	0.0042 (4)	−0.0087 (4)
C26	0.0199 (5)	0.0272 (5)	0.0207 (5)	0.0011 (4)	0.0040 (4)	−0.0037 (4)
O1W	0.0213 (4)	0.0271 (4)	0.0182 (3)	0.0037 (3)	0.0037 (3)	0.0042 (3)

### Geometric parameters (Å, °)

**Table:**

O7—C7	1.2336 (12)	C21—C26	1.3978 (14)
O1W—H1W	0.917 (17)	C22—C23	1.3850 (15)
O1W—H2W	0.851 (17)	C23—C24	1.3816 (17)
N1—C2	1.3394 (13)	C24—C25	1.3874 (17)
N1—C6	1.3455 (13)	C25—C26	1.3841 (15)
N8—C7	1.3515 (13)	C2—H2	0.9500
N8—N9	1.3868 (11)	C4—H4	0.9500
N9—C10	1.2854 (13)	C5—H5	0.9500
N8—H8	0.882 (14)	C6—H6	0.9500
C2—C3	1.3960 (14)	C10—H10	0.9500
C3—C7	1.4960 (13)	C12—H12	0.9500
C3—C4	1.3901 (13)	C13—H13A	0.9800
C4—C5	1.3895 (14)	C13—H13C	0.9800
C5—C6	1.3789 (14)	C13—H13B	0.9800
C10—C11	1.4537 (14)	C22—H22	0.9500
C11—C12	1.3501 (14)	C23—H23	0.9500
C11—C13	1.5008 (15)	C24—H24	0.9500
C12—C21	1.4630 (14)	C25—H25	0.9500
C21—C22	1.4027 (14)	C26—H26	0.9500
			
O1W···C10^i^	3.3781 (13)	C23···H4^v^	2.8800
O1W···O7	2.9089 (10)	C24···H4^v^	3.0300
O1W···N9	3.2233 (11)	C24···H5^viii^	3.0900
O1W···C13	3.3815 (14)	C25···H5^viii^	2.9100
O1W···N1^ii^	2.9033 (11)	C26···H5^viii^	2.7100
O1W···N8^i^	2.8925 (12)	H1W···C6^ii^	3.040 (17)
O1W···C4^i^	3.3753 (12)	H1W···N1^ii^	1.987 (17)
O7···O1W	2.9089 (10)	H1W···C2^ii^	2.776 (17)
O7···C10^i^	3.2830 (13)	H2···O7	2.5200
O7···N9	2.7031 (10)	H2···O7^ii^	2.5400
O7···C2^ii^	3.4021 (12)	H2W···C7	2.994 (17)
O1W···H10^i^	2.5900	H2W···N9	2.507 (17)
O1W···H13B	2.5500	H2W···O7	2.161 (17)
O1W···H8^i^	2.029 (14)	H2W···H10^i^	2.5600
O1W···H4^i^	2.7700	H2W···H13B	2.4800
O7···H2^ii^	2.5400	H2W···H8^i^	2.35 (2)
O7···H10^i^	2.7400	H4···N8	2.6600
O7···H2	2.5200	H4···H23^v^	2.5800
O7···H2W	2.161 (17)	H4···C24^v^	3.0300
O7···H26^iii^	2.5400	H4···H8	2.2100
N1···O1W^ii^	2.9033 (11)	H4···O1W^iv^	2.7700
N8···O1W^iv^	2.8925 (12)	H4···C23^v^	2.8800
N9···O1W	3.2233 (11)	H5···C24^ix^	3.0900
N9···O7	2.7031 (10)	H5···C25^ix^	2.9100
N1···H22^iv^	2.9500	H5···C26^ix^	2.7100
N1···H1W^ii^	1.987 (17)	H5···C22^ix^	2.9500
N8···H4	2.6600	H5···C21^ix^	2.7100
N9···H13B	2.4700	H6···H24^vii^	2.4400
N9···H2W	2.507 (17)	H8···H2W^iv^	2.35 (2)
C2···O7^ii^	3.4021 (12)	H8···H4	2.2100
C4···C24^v^	3.5844 (15)	H8···C4	2.640 (14)
C4···C13^iv^	3.5229 (15)	H8···H10	2.1000
C4···O1W^iv^	3.3753 (12)	H8···O1W^iv^	2.029 (14)
C5···C6^vi^	3.4847 (15)	H10···H8	2.1000
C5···C13^iv^	3.5988 (16)	H10···H2W^iv^	2.5600
C6···C5^vi^	3.4847 (15)	H10···H12	2.2400
C10···C21^v^	3.5585 (14)	H10···O1W^iv^	2.5900
C10···O7^iv^	3.2830 (13)	H10···O7^iv^	2.7400
C10···O1W^iv^	3.3781 (13)	H12···H26	2.3900
C10···C22^v^	3.5673 (15)	H12···H10	2.2400
C13···O1W	3.3815 (14)	H13A···C21	2.8800
C13···C5^i^	3.5988 (16)	H13A···C22	2.6400
C13···C4^i^	3.5229 (15)	H13A···H22	2.1800
C13···C22	3.1004 (15)	H13B···O1W	2.5500
C21···C10^v^	3.5585 (14)	H13B···N9	2.4700
C22···C13	3.1004 (15)	H13B···H25^iii^	2.3800
C22···C10^v^	3.5673 (15)	H13B···H2W	2.4800
C24···C4^v^	3.5844 (15)	H13C···H22	2.3400
C2···H13C^iv^	3.0700	H13C···C2^i^	3.0700
C2···H1W^ii^	2.776 (17)	H13C···C5^i^	3.0400
C3···H13C^iv^	2.8500	H13C···C3^i^	2.8500
C4···H13C^iv^	2.8300	H13C···C4^i^	2.8300
C4···H8	2.640 (14)	H22···H13A	2.1800
C5···H13C^iv^	3.0400	H22···H13C	2.3400
C6···H1W^ii^	3.040 (17)	H22···N1^i^	2.9500
C6···H22^iv^	3.0000	H22···C6^i^	3.0000
C6···H24^vii^	3.0600	H22···C11	2.9300
C7···H26^iii^	2.8000	H22···C13	2.5400
C7···H2W	2.994 (17)	H23···H4^v^	2.5800
C11···H22	2.9300	H24···C6^x^	3.0600
C13···H22	2.5400	H24···H6^x^	2.4400
C21···H13A	2.8800	H25···H13B^xi^	2.3800
C21···H5^viii^	2.7100	H26···O7^xi^	2.5400
C22···H13A	2.6400	H26···C7^xi^	2.8000
C22···H5^viii^	2.9500	H26···H12	2.3900
			
H1W—O1W—H2W	100.8 (15)	C3—C2—H2	118.00
C2—N1—C6	117.12 (8)	N1—C2—H2	118.00
N9—N8—C7	118.59 (8)	C3—C4—H4	120.00
N8—N9—C10	114.07 (8)	C5—C4—H4	120.00
N9—N8—H8	117.5 (9)	C4—C5—H5	121.00
C7—N8—H8	123.6 (8)	C6—C5—H5	121.00
N1—C2—C3	123.47 (9)	C5—C6—H6	118.00
C4—C3—C7	124.28 (9)	N1—C6—H6	118.00
C2—C3—C4	118.11 (9)	N9—C10—H10	119.00
C2—C3—C7	117.61 (8)	C11—C10—H10	119.00
C3—C4—C5	119.01 (9)	C21—C12—H12	115.00
C4—C5—C6	118.57 (9)	C11—C12—H12	115.00
N1—C6—C5	123.71 (9)	C11—C13—H13A	109.00
N8—C7—C3	115.18 (8)	C11—C13—H13C	109.00
O7—C7—N8	123.51 (9)	H13A—C13—H13B	109.00
O7—C7—C3	121.30 (8)	H13A—C13—H13C	109.00
N9—C10—C11	121.40 (9)	H13B—C13—H13C	109.00
C10—C11—C12	116.59 (9)	C11—C13—H13B	109.00
C10—C11—C13	118.24 (9)	C23—C22—H22	120.00
C12—C11—C13	125.17 (9)	C21—C22—H22	120.00
C11—C12—C21	129.50 (9)	C22—C23—H23	120.00
C22—C21—C26	117.45 (9)	C24—C23—H23	120.00
C12—C21—C22	124.28 (9)	C25—C24—H24	120.00
C12—C21—C26	118.26 (9)	C23—C24—H24	120.00
C21—C22—C23	120.93 (10)	C24—C25—H25	120.00
C22—C23—C24	120.60 (11)	C26—C25—H25	120.00
C23—C24—C25	119.30 (10)	C21—C26—H26	119.00
C24—C25—C26	120.24 (10)	C25—C26—H26	119.00
C21—C26—C25	121.33 (10)		
			
C6—N1—C2—C3	−1.84 (15)	C4—C5—C6—N1	0.42 (16)
C2—N1—C6—C5	0.88 (15)	N9—C10—C11—C12	−177.04 (9)
C7—N8—N9—C10	179.25 (9)	N9—C10—C11—C13	2.96 (15)
N9—N8—C7—O7	−2.13 (15)	C10—C11—C12—C21	178.57 (9)
N9—N8—C7—C3	179.25 (8)	C13—C11—C12—C21	−1.43 (17)
N8—N9—C10—C11	179.05 (9)	C11—C12—C21—C22	−22.57 (17)
N1—C2—C3—C4	1.46 (15)	C11—C12—C21—C26	156.91 (10)
N1—C2—C3—C7	−178.27 (9)	C12—C21—C22—C23	175.37 (10)
C2—C3—C4—C5	−0.06 (13)	C26—C21—C22—C23	−4.11 (15)
C7—C3—C4—C5	179.65 (9)	C12—C21—C26—C25	−176.07 (10)
C2—C3—C7—O7	−23.90 (14)	C22—C21—C26—C25	3.45 (15)
C2—C3—C7—N8	154.76 (9)	C21—C22—C23—C24	1.54 (17)
C4—C3—C7—O7	156.39 (10)	C22—C23—C24—C25	1.83 (17)
C4—C3—C7—N8	−24.94 (14)	C23—C24—C25—C26	−2.50 (17)
C3—C4—C5—C6	−0.81 (15)	C24—C25—C26—C21	−0.20 (16)

Symmetry codes: (i) −*x*, *y*−1/2, −*z*+1/2; (ii) −*x*, −*y*+1, −*z*; (iii) *x*, −*y*+3/2, *z*−1/2; (iv) −*x*, *y*+1/2, −*z*+1/2; (v) −*x*, −*y*+1, −*z*+1; (vi) −*x*−1, −*y*+1, −*z*; (vii) *x*−1, *y*, *z*−1; (viii) *x*+1, −*y*+3/2, *z*+1/2; (ix) *x*−1, −*y*+3/2, *z*−1/2; (x) *x*+1, *y*, *z*+1; (xi) *x*, −*y*+3/2, *z*+1/2.

### Hydrogen-bond geometry (Å, °)

**Table:**

*D*—H···*A*	*D*—H	H···*A*	*D*···*A*	*D*—H···*A*
O1W—H1W···N1^ii^	0.917 (17)	1.987 (17)	2.9033 (11)	177.3 (16)
O1W—H2W···O7	0.851 (17)	2.161 (17)	2.9089 (10)	146.5 (15)
O1W—H2W···N9	0.851 (17)	2.507 (17)	3.2233 (11)	142.5 (14)
N8—H8···O1W^iv^	0.882 (14)	2.029 (14)	2.8925 (12)	166.0 (13)
C2—H2···O7^ii^	0.95	2.54	3.4021 (12)	151
C10—H10···O1W^iv^	0.95	2.59	3.3781 (13)	140
C13—H13B···O1W	0.98	2.55	3.3815 (14)	143
C26—H26···O7^xi^	0.95	2.54	3.4771 (13)	170
C13—H13C···Cg1^i^	0.98	2.72	3.5630 (13)	144
C5—H5···Cg2^ix^	0.95	2.57	3.4378 (11)	152

Symmetry codes: (ii) −*x*, −*y*+1, −*z*; (iv) −*x*, *y*+1/2, −*z*+1/2; (xi) *x*, −*y*+3/2, *z*+1/2; (i) −*x*, *y*−1/2, −*z*+1/2; (ix) *x*−1, −*y*+3/2, *z*−1/2.
